# Notes from Practice

**Published:** 1877-11

**Authors:** 


### NOTES FROM PRACTICE.

---

*Practical Hints in Dental Mechanism.*—Your flasks for Vulcanite give you trouble. Bolts break, or threads strip; the hot flask is difficult to handle; the dirt peculiar to this work is specially adherent,—no amount of scrubbing seems to move it;—the pressure on the blocks, cracks or separates them, and in frail cases with poor plaster, the model itself is injured. To avoid these troubles and vexations, get a flask press, the older forms of the celluloid press—a stirrup and screw—is an excellent apparatus for the purpose, and with this and a block of spring rubber—the kind of which car springs, &c. are made—the difficulties are in most part avoided. Place the flask after packing, in the press, and placing the block of rubber, which for this purpose should be one and one-half inches square and half or three-fourths of an inch thick, on the top of the flask, and the center-piece, or disk of iron on the top of this and then turn the screw gently in position. Put it into your boiler of water. The rubber will soon be softened by the heat, when the screw may be turned down, using no more pressure than the finger and thumb—the wood handle of the press ought to be removed—until the rubber under the disk is flattened out considerably. You may now go off and do what else you have to do, your flask is closing gently, surely, automatically. The pressure is so steady and gradual as to force nothing unduly, and all handling of the flask is avoided.

---

*Broaches for Pulp Canals.*—For those who prefer or are compelled to make nerve broaches for themselves. nothing is better—perhaps nothing so good—as a piece of *piano wire.* It is of good steel, drawn to a spring temper, and is exceedingly strong and tough. It may be gotten of different sizes and filed down to suit. We have found these excellent for broaches and also as pluggers for filling the roots. They may be held in a broach holder, or mounted in a light wooden handle,—the latter is best, giving lightness and avoiding the changing the other would necessitate. By cutting with a chisel a series of barbs on one side they may be used to rapidly cut out or enlarge the root canals.

*A Case in Practice.*—In excavating a cavity in a superior bicuspid, accidental exposure of the pulp occurred, with no pain of consequence. The point of exposure was very small.

A drop of gutta-percha dissolved in chloroform was applied, the application being easy on account of the loss of the adjacent tooth. Over this was placed a filling of os-artificiel, when pain instantly set in, and increased in intensity, until in an hour the whole face was in agony, the eye suffused, and no yielding to counter irritants manifested. The filling was removed, but with no abatement of the pain; nor did the application of the traditional remedies in the slightest degree moderate the suffering which was now intolerable. Arsenic was applied, and in five minutes all severe pain was gone, and the patient comparatively comfortable. The patient was an excessively nervous, overworked dyspeptic.

QUERY.—What else ought to have been done, if any thing?

---

*Anæsthetic effects of Cold Water.*—A writer in the *Medical and Surgical Reporter* gives it as a fact that the hypodermic injection of a few drops of cold water produces remarkable results in the relief of pain in rheumatism and other acute diseases. A short time ago a statement was currently published, to the effect that a physician had accidentally substituted water for a solution of morphia, with the happiest results in sciatica. This writer, having a case on his hands where the patient, suffering with phthisis, was in the habit of taking hypodermics of morphia regularly for the refief of cough, substituted water on one occasion only, which was not a success. No amount of evasion would convince her that a mistake had not been made, and he was glad at the expiration of half an hour to get rid of his patient's importunities by using the morphia itself. "Ah!" said she, "that feels something like. That other was no better than water."

---

